# Clinicoradiological Features and Surgical Outcomes of Cauda Equina Neuroendocrine Tumors: A Single-Center Retrospective Study

**DOI:** 10.3390/jcm15114332

**Published:** 2026-06-03

**Authors:** Mehmet Tiryaki, Bekir Can Kendirlioğlu, Halit Alioglu, Omar Alomari, Ayca Ceylan Akgul, Serife Altunbay, Ibrahim Ilker Oz, Zuhal Kus Silav, Hikmet Turan Suslu

**Affiliations:** 1 Department of Neurosurgery, Dr. Lutfi Kirdar Kartal Training and Research Hospital, University of Health Sciences, Istanbul 34890, Türkiye; can.kendirlioglu@gmail.com (B.C.K.); okailykhaled@gmail.com (H.A.);; 2 Hamidiye International School of Medicine, University of Health Sciences, Istanbul 34668, Türkiye; dromari2001@gmail.com; 3 Department of Radiology, Dr. Lutfi Kirdar Kartal Training and Research Hospital, University of Health Sciences, Istanbul 34890, Türkiye; 4 Department of Pathology, Dr. Lutfi Kirdar Kartal Training and Research Hospital, University of Health Sciences, Istanbul 34890, Türkiye

**Keywords:** CENET, cauda equina, intradural extramedullary tumor, eccentric vessel sign, microsurgical resection, surgical outcomes

## Abstract

**Background/Objectives:** Cauda equina neuroendocrine tumors (CENETs) are rare neuroendocrine tumors that predominantly arise in the cauda equina and filum terminale region. Due to their nonspecific clinical and radiological features, preoperative diagnosis remains challenging, and available data are limited to small case series. **Methods:** This retrospective single-center study included nine patients who underwent surgical treatment for histopathologically confirmed CENETs between 2014 and 2025. Clinical presentation, radiological findings, surgical management, histopathological features, and postoperative outcomes were analyzed. **Results:** The mean age was 51.2 years, with a slight male predominance. Pain was the most common presenting symptom (77.8%), followed by radiculopathy and sensory disturbances. All tumors were intradural and extramedullary, predominantly located in the lumbosacral region. Radiologically, all lesions were isointense on T1-weighted imaging and demonstrated predominantly homogeneous contrast enhancement. Vascular imaging features, including flow voids (55.6%), eccentric vessel sign (66.7%), and tadpole sign (44.4%), were frequently observed. Gross total resection was achieved in all patients, with no neurological deterioration or major complications. Over a mean follow-up period of 59.6 months, no tumor recurrence was detected. **Conclusions:** Cauda equina neuroendocrine tumors are rare but surgically curable tumors with excellent prognosis. Although preoperative diagnosis remains difficult, recognition of characteristic vascular imaging features may improve diagnostic accuracy. Gross total resection remains the cornerstone of treatment, providing durable disease control with minimal morbidity.

## 1. Introduction

Cauda equina neuroendocrine tumors (CENETs) are rare neuroendocrine tumors arising from neural crest–derived paraganglionic cells and are most commonly located in the cauda equina and filum terminale region [1,2]. According to the 2022 World Health Organization classification of central nervous system tumors, CENETs are categorized as distinct, typically low-grade neoplasms with characteristic histopathological and immunohistochemical features [3]. CENETs may arise in various anatomical locations throughout the body, with more than 90% originating in the head and neck region, where they are typically associated with parasympathetic structures, including the carotid body (chemodectomas), vagus nerve, and jugulotympanic region [4,5,6]. In contrast, spinal involvement is rare and predominantly affects the cauda equina and filum terminale [7,8]. Despite their generally benign clinical course, these tumors often present with nonspecific symptoms such as low back pain and radiculopathy, frequently leading to diagnostic challenges [9,10]. The differential diagnosis of lesions in this region is typically dominated by ependymomas and nerve sheath tumors, and preoperative differentiation from these intradural extramedullary neoplasms remains difficult due to overlapping radiological features [11,12].

Due to the rarity of CENETs, the available literature remains largely limited to case reports and small series, leaving comprehensive data regarding their clinical behavior and long-term surgical outcomes scarce. To address this gap in the literature, the primary purpose of this study is to systematically evaluate the clinical presentation, radiological characteristics, and surgical management of CENETs within a single-center cohort. Specifically, this study seeks to determine which distinct clinicoradiological features are most predictive of achieving a gross total resection and to evaluate the subsequent long-term recurrence-free survival rates based on the degree of surgical clearance. By analyzing the long-term outcomes of this single-center series of nine consecutive patients, we aim to provide robust clinical insights that can refine preoperative planning, optimize surgical strategies, and guide postoperative surveillance protocols for these rare vascular tumors.

## 2. Materials and Methods

### 2.1. Study Design and Patient Selection

This retrospective, single-center study evaluated patients evaluated and managed for spinal masses at our institution between 2014 and 2025. To establish the study cohort, specific inclusion and exclusion criteria were applied. The inclusion criteria required patients to have a histopathologically confirmed diagnosis of spinal CENET and to have undergone primary surgical resection at our center during the specified study period. Conversely, patients were excluded from the final analysis based on the following criteria: the presence of concurrent or synchronous spinal tumors of different etiologies, management via conservative non-surgical modalities, incomplete institutional clinical data records, or a total lack of postoperative radiological follow-up data. Potential cases were identified through a comprehensive review of institutional medical records, surgical databases, and pathology archives. For all matching individuals, comprehensive data regarding demographic characteristics, clinical presentation, radiological findings, histopathological features, surgical details, and long-term postoperative outcomes were collected and analyzed. Applying these strict eligibility criteria resulted in a final cohort of nine consecutive patients who met all requirements and were included in the final analysis.

### 2.2. Radiological Evaluation

All patients underwent preoperative magnetic resonance imaging (MRI) of the spine. Radiological assessment was performed using preoperative MRI scans retrieved from the institutional imaging archive. The following parameters were evaluated: tumor localization and segmental level, anatomical compartment (intradural extramedullary or extradural), and tumor dimensions, including craniocaudal length, anteroposterior diameter, and transverse width. Signal characteristics on T1- and T2-weighted sequences and the pattern of contrast enhancement were recorded. In addition, secondary imaging features were assessed, including the presence of flow voids, cystic components, hemorrhagic features, hemorrhagic cap sign, eccentric vessel sign, dumbbell configuration, tadpole sign, and osseous remodeling.

### 2.3. Histopathological and Immunohistochemical Analysis

All tumor specimens were obtained during surgical resection and subjected to histopathological examination. The diagnosis of spinal CENET was established based on characteristic morphological features, including the presence of a nested (“zellballen”) growth pattern composed of chief cells surrounded by sustentacular cells. Immunohistochemical analysis was performed using standard protocols. The expression of neuroendocrine markers, including chromogranin and synaptophysin, as well as S100 protein, was evaluated. The Ki-67 proliferation index was also recorded for each case when available. All histopathological and immunohistochemical data were retrieved from institutional pathology reports.

### 2.4. Surgical Management

All patients underwent microsurgical tumor resection using standard posterior spinal approaches. The surgical approach was selected according to tumor location and extent and included total laminectomy, hemilaminectomy, or laminoplasty. The relationship of the tumor to adjacent neural structures, including the filum terminale and nerve roots, was assessed intraoperatively, and preservation or sacrifice was documented. The extent of tumor resection was determined based on intraoperative findings, surgical reports, and postoperative imaging and was categorized as gross total resection (GTR), subtotal resection (STR), or biopsy.

### 2.5. Functional Outcome Assessment

Neurological status was evaluated using the Modified McCormick Scale (MMcCS). Postoperative neurological status was assessed in the early postoperative period. Functional outcome was categorized as improved, stable, or worsened based on postoperative neurological evaluation.

### 2.6. Statistical Analysis

Descriptive statistics were used to summarize all study variables. Continuous data (e.g., age, tumor size, follow-up duration) are presented as means with ranges, while categorical data (clinical symptoms, radiological signs, McCormick grades) are reported as absolute frequencies and percentages. Given the sample size, formal inferential testing was not performed. Data were managed and analyzed using [R v4.3.2].

### 2.7. Ethical Approval

This study was conducted in accordance with the principles of the Declaration of Helsinki. Ethical approval was obtained from the Scientific Research Ethics Committee of Kartal Dr. Lütfi Kırdar City Hospital (Approval No: 2026/01.09.25/46; Date: 24 February 2026).

## 3. Results

### 3.1. Demographic and Clinical Characteristics

Nine patients with histologically confirmed CENETs were included (Table 1). The mean age at presentation was 51.2 years (range: 38–67), with a slight male predominance (5 males, 4 females) (Table 2). Comorbidities were present in 22.2% of patients, including diabetes mellitus and chronic hepatitis B. Clinically, pain was the most common presenting symptom (77.8%), followed by radiculopathy and hypoesthesia (each 44.4%). No patients reported paresthesia, and only one patient (11.1%) presented with incontinence. The mean symptom duration prior to diagnosis was 7.4 months (range: 3–24 months). All patients were neurologically well preserved at presentation, with 55.6% classified as McCormick Grade I and 44.4% as Grade II, and no patients demonstrating advanced neurological deficits.

### 3.2. Radiological Findings

Magnetic resonance imaging demonstrated intradural extramedullary localization in all cases (100%), predominantly in the lumbosacral region, most frequently at the L3 level (33.3%). Mean tumor dimensions were 23.3 mm in the craniocaudal axis, 11.4 mm anteroposteriorly, and 13.9 mm transversely. All lesions were isointense on T1-weighted imaging, while T2 signal intensity was variable, appearing isointense in 55.6% and hyperintense in 44.4% of cases. Post-contrast imaging demonstrated homogeneous enhancement in 66.7% and heterogeneous enhancement in 33.3%.

**Figure 1:**
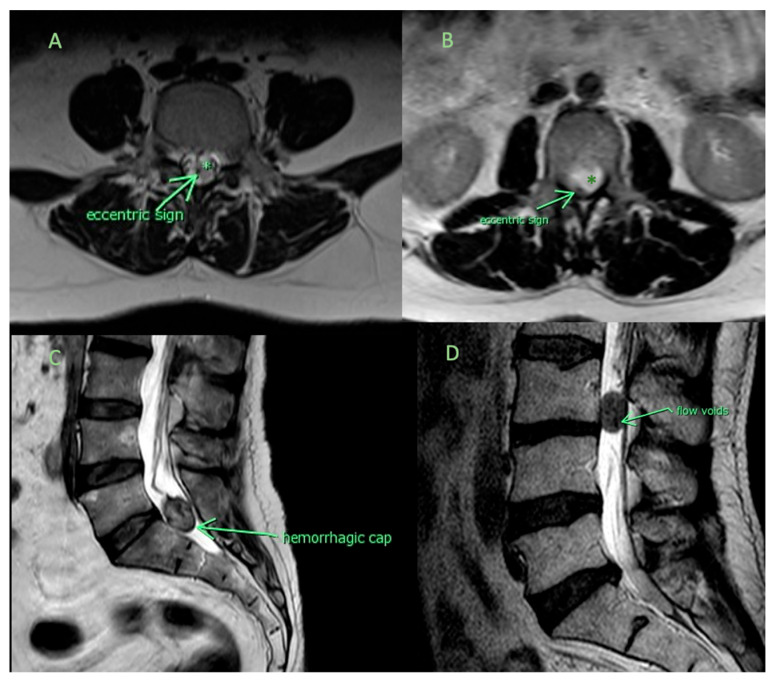
Representative magnetic resonance imaging findings of CENETs. Axial T2-weighted images (**A**,**B**) demonstrate the “eccentric vessel sign” (arrows), characterized by a serpiginous vascular structure located eccentrically within the tumor. Sagittal T2-weighted image (**C**) shows a lesion with a “hemorrhagic cap sign” (arrow), representing a hypointense rim due to hemosiderin deposition. Sagittal T2-weighted image (**D**) demonstrates prominent intralesional and peritumoral flow voids (arrowheads), consistent with the hypervascular nature of these tumors.

Notably, characteristic imaging features were frequently observed, including flow voids and hemorrhagic components (each 55.6%), hemorrhagic cap sign (44.4%), eccentric vessel sign (66.7%), and tadpole sign (44.4%). (Figure 1) Cystic elements were present in 22.2% of cases. No tumors demonstrated a dumbbell configuration or osseous remodeling.

### 3.3. Surgical Management and Early Outcomes

The mean time from diagnosis to surgery was 27 days (Median 3-913) (Table 3). Surgical approaches included total laminectomy (44.4%), hemilaminectomy (44.4%), and laminoplasty (11.1%). Gross total resection was achieved in all patients (100%), with complete preservation of neural structures in every case. Early postoperative outcomes were favorable, with no cases of neurological deterioration; 44.4% of patients demonstrated neurological improvement, while 55.6% remained stable. No intraoperative or early postoperative complications were observed. One patient had significantly elevated preoperative blood pressure levels, which were effectively managed in the postoperative period with beta-blocker therapy, without further clinical sequelae.

### 3.4. Histopathological Findings and Long-Term Outcomes

Histopathological examination confirmed the diagnosis of CENET in all cases (Table 3). Immunohistochemical analysis demonstrated universal positivity for chromogranin and synaptophysin, with S100 positivity in 88.9% of cases. The Ki-67 proliferation index was low, with a mean value of 7.0% (range: 2–15%). The postoperative course was uneventful, with a mean hospital stay of 4.3 days. No patients required adjuvant therapy. Over a mean follow-up period of 59.6 months (range: 12–134 months), no tumor recurrences were observed.

## 4. Illustrative Cases

Case 1

**Figure 2:**
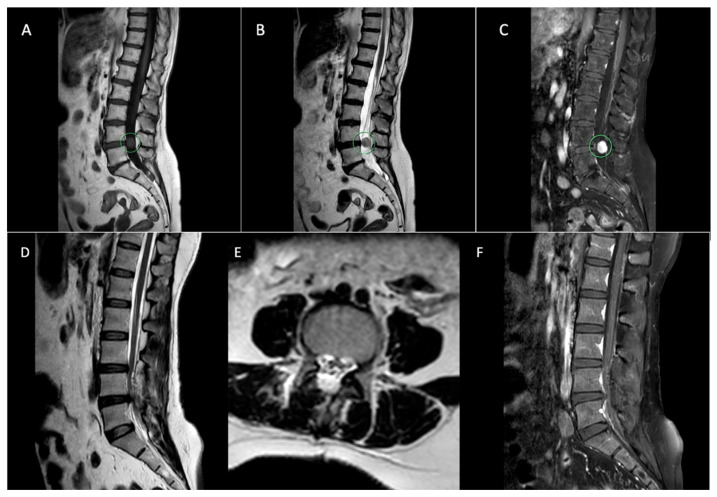
Preoperative and postoperative MRI findings of Case 1. Sagittal preoperative T1-, T2-, and contrast-enhanced T1-weighted images (**A**–**C**) demonstrate a well-circumscribed intradural extramedullary lesion at the L4–5 level, appearing isointense on both T1- and T2-weighted sequences with homogeneous contrast enhancement (circles). Postoperative sagittal and axial T2- and contrast-enhanced T1-weighted images (**D**–**F**) confirm gross total resection with no evidence of residual tumor.

A 47-year-old female presented with low back pain and no significant past medical history. Preoperative neurological status was McCormick Grade I. Magnetic resonance imaging revealed an intradural extramedullary lesion at the L4-5 level (09 × 10 × 11 mm^3^), which was isointense on both T1- and T2-weighted sequences and demonstrated homogeneous contrast enhancement (Figure 2A–C). Gross total resection was achieved (Figure 2D–F). Histopathological examination confirmed CENET with a Ki-67 proliferation index of 6%. The patient remained neurologically intact postoperatively (McCormick Grade I), showed clinical improvement, and no recurrence was observed at 24 months of follow-up.

Case 4

**Figure 3:**
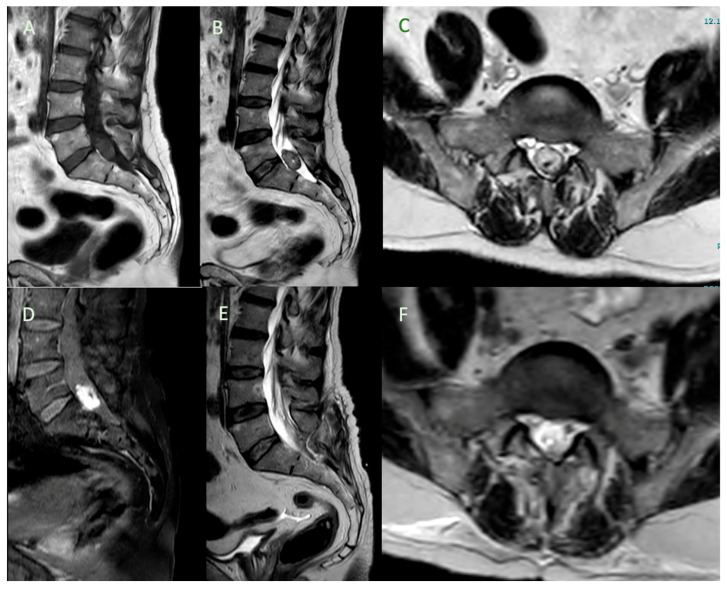
Preoperative and postoperative MRI findings of Case 4. Sagittal and axial preoperative T1-, T2-, and contrast-enhanced T1-weighted images (**A**–**D**) demonstrate an intradural extramedullary lesion at the S1 level with heterogeneous enhancement. Postoperative images (**E**,**F**) confirm gross total resection without residual lesion.

A 58-year-old male presented with chronic low back pain. His medical history was notable for hypertension. Preoperative neurological examination revealed McCormick Grade I status. MRI demonstrated an intradural extramedullary lesion at the S1 level (26 × 15 × 18 mm ^3^) with heterogeneous contrast enhancement (Figure 3A–D). Gross total resection was achieved (Figure 3E,F). Histopathological examination confirmed CENET with a Ki-67 proliferation index of 4%.

The postoperative course was uneventful. Notably, the patient demonstrated stable baseline hemodynamics preoperatively, but developed a severe, transient hypertensive spike (up to 210/180 mmHg) intraoperatively during the direct microsurgical manipulation and mobilization of the tumor mass. This surge was effectively managed in real-time with an acute titration of short-acting intravenous beta-blocker therapy by the neuroanesthesia team, along with an adjustment of anesthetic depth. Following tumor removal, blood pressure normalized rapidly without further sequelae. The patient remained neurologically stable (McCormick Grade I), and no recurrence was observed at 35 months of follow-up.

Case 9

**Figure 4:**
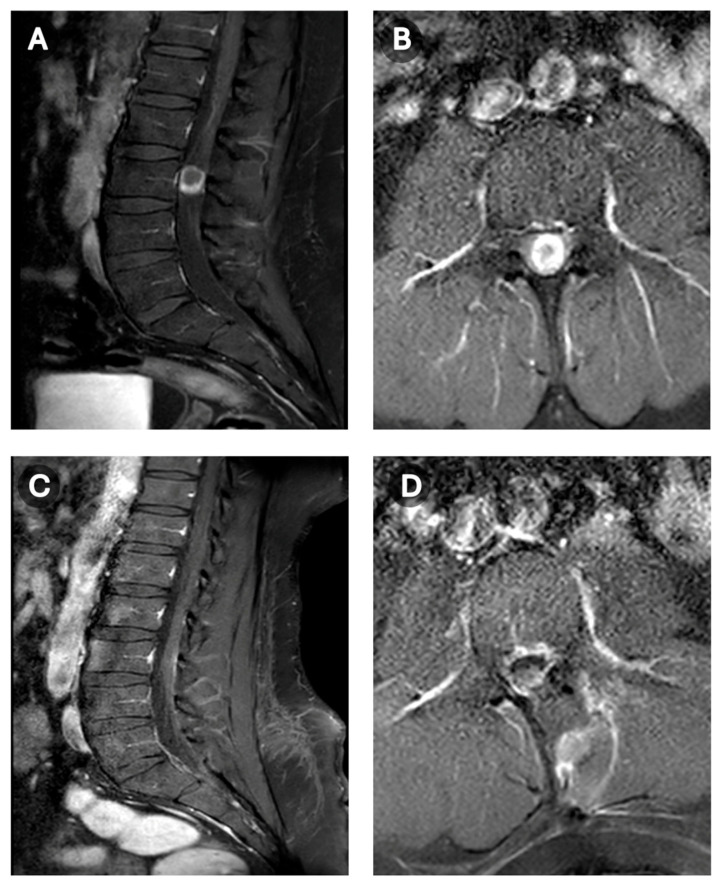
Preoperative and postoperative MRI findings of Case 9. Sagittal and axial preoperative contrast-enhanced T1-weighted images (**A**,**B**) demonstrate an intradural extramedullary lesion at the L3 level with heterogeneous enhancement. Postoperative images (**C**,**D**) confirm gross total resection.

**Figure 5:**
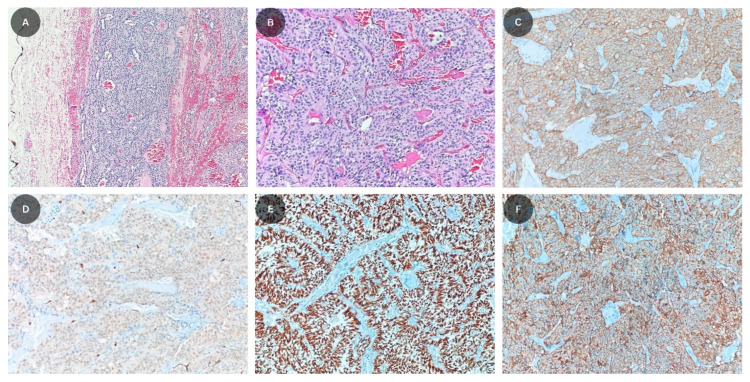
Histopathological features of Cauda Equina Neuroendocrine Tumor. (**A**) Hematoxylin and eosin (H&E) photomicrograph (×100 HPF) demonstrating a well-encapsulated neoplasm composed of organoid nests of tumor cells (zellballen pattern) separated by prominent fibrovascular septa, consistent with the characteristic histomorphology of Cauda Equina Neuroendocrine Tumor. (**B**) Hematoxylin and eosin (H&E) photomicrograph (×200 HPF) demonstrating uniform epithelioid tumor cells (chief cells) with round nuclei, finely granular (“salt-and-pepper”) chromatin, and abundant granular eosinophilic cytoplasm, arranged in a characteristic nested architecture. (**C**) Synaptophysin immunohistochemistry (×200 HPF) demonstrating diffuse and strong cytoplasmic positivity in chief tumor cells, supporting neuroendocrine differentiation characteristic of Cauda Equina Neuroendocrine Tumor. (**D**) S100 immunohistochemistry (×200 HPF) demonstrating positive staining in perilobular sustentacular cells surrounding the nests of chief cells, supporting the characteristic biphasic cellular architecture of Cauda Equina Neuroendocrine Tumor. (**E**) Pan-cytokeratin immunohistochemistry (×200 HPF) demonstrating diffuse and strong cytoplasmic expression in epithelioid tumor cells (chief cells), supporting the characteristic epithelial neuroendocrine differentiation of cauda equina Cauda Equina Neuroendocrine Tumor. (**F**) Chromogranin immunohistochemistry (×200 HPF) demonstrating diffuse and strong cytoplasmic reactivity in chief tumor cells, supporting the neuroendocrine differentiation characteristic of Cauda Equina Neuroendocrine Tumor.

A 45-year-old male presented with low back pain and paresthesia, without any significant past medical history. Preoperative neurological status was McCormick Grade II. Magnetic resonance imaging revealed an intradural extramedullary lesion at the L3 level (20 × 12 × 13 mm^3^), isointense on T1-weighted images and hyperintense on T2-weighted sequences, with heterogeneous contrast enhancement (Figure 4A,B). Gross total resection was achieved (Figure 4C,D). Histopathological and immunohistochemical examination confirmed a Cauda Equina Neuroendocrine Tumor (CENET) with a low Ki-67 proliferation index of 2% (Figure 5A–F). Postoperatively, the patient improved neurologically to McCormick Grade I, and no recurrence was detected at 18 months of follow-up.

## 5. Discussion

Primary CENETs are rare, slow-growing, benign intradural extramedullary tumors that most commonly arise in the cauda equina and filum terminale, accounting for approximately up to 3.5% of lesions in this region [4,13,14,15]. According to the World Health Organization (WHO), they are classified as grade I neoplasms originating from neuroectodermal paraganglionic cells containing biogenic amine and peptide-rich secretory granules [5,6]. Although the majority exhibit indolent behavior, a small subset (2.4–14%) may demonstrate malignant potential [16,17]. Functionally, these tumors are predominantly non-secretory, while catecholamine-producing variants remain rare [17]. Approximately 80–90% of CENETs arise in the head and neck region, particularly from the carotid body and jugulotympanic structures, and may rarely disseminate to other anatomical sites. In contrast, CENETs are exceptionally uncommon [18,19].

Since the first description of a filum terminale CENET by Lerman in 1972 [1], these tumors have long been recognized as primarily affecting the lumbosacral spine, although their exact prevalence is still uncertain [7]. This classic anatomical predilection was perfectly mirrored in our cohort, where 100% of the cases demonstrated an intradural extramedullary localization restricted to the lumbosacral region, occurring most frequently at the L3 level (33.3%). Because of this location, the clinical presentation of CENET is typically nonspecific, most commonly manifesting as low back pain and radiculopathy [9,20]. In our series, pain was the predominant initial symptom, affecting 77.8% of the patients, followed by radiculopathy and hypoesthesia in 44.4% of cases, respectively. These findings are highly consistent with previous reports [20,21] and reflect the slow-growing, indolent nature of these vascular lesions as they progressively compress the nerve roots of the cauda equina. This slow clinical evolution is further supported by the relatively prolonged symptom duration observed in our cohort prior to diagnosis, which averaged 7.4 months and spanned up to 24 months [14]. Despite this prolonged compression, the slow progression allowed the majority of our patients to remain neurologically well preserved at presentation, with 55.6% classified as McCormick Grade I and 44.4% as Grade II.

Due to these nonspecific features, preoperative diagnosis remains challenging, and these lesions are frequently misinterpreted as more common intradural tumors such as schwannomas, ependymomas, meningiomas, or solitary metastases [12,22,23]. Radiological evaluation plays a key role in the preoperative assessment of CENETs, although imaging findings are often not specific. As emphasized by Gelabert-González et al. and Turk et al., these tumors frequently mimic other intradural extramedullary lesions, particularly schwannomas and ependymomas [9,23]. In our series, all lesions were isointense on T1-weighted imaging and predominantly demonstrated homogeneous contrast enhancement, findings that are consistent with previous reports, including those by Yin et al. and Fiorini et al. [14,21]. Notably, the eccentric vessel sign was observed in 66.7% of our patients. In line with this, Ajmera et al. reported this sign in approximately 80% of cases and suggested it as a potentially reliable imaging marker for CENETs [12]. Similarly, Yi et al. described the “tadpole sign” as another vascular imaging feature associated with these tumors [24]. In our cohort, the tadpole sign was observed in 44.4% of cases, which is lower than the rate reported by Yi et al., possibly reflecting differences in tumor size and sample characteristics. Additional findings such as flow voids (55.6%) further support the hypervascular nature of these lesions. Despite these imaging clues, accurate preoperative diagnosis remains challenging due to substantial radiological overlap with more common spinal tumors. Surgical resection remains the cornerstone of treatment for CENETs. As highlighted by Yang et al. and Fiorini et al., gross total resection (GTR) is generally considered curative and is associated with excellent clinical outcomes [7,14]. In the present series, GTR was achieved in all patients (100%), with no cases of neurological deterioration or major postoperative complications, further supporting the safety and effectiveness of microsurgical resection. Similarly, Gelabert-González et al. reported favorable outcomes and very low recurrence rates following complete tumor removal [9].

The highly vascular nature of these tumors, as also emphasized in previous studies, necessitates meticulous surgical technique, particularly in relation to the filum terminale and adjacent nerve roots [7,14].

These lesions are frequently supplied by prominent feeding vessels arising along the filum terminale or adjacent nerve roots, which can cause significant hemorrhage if breached prematurely [25]. To manage this hypervascularity safely, surgery must prioritize early identification and controlled bipolar coagulation of these primary vascular pedicles at the upper and lower poles of the tumor before attempting tumor mobilization. Furthermore, because these lesions slowly expand within the intradural extramedullary space, they tend to develop dense arachnoidal adhesions to the surrounding mobile roots of the cauda equina. This requires a patient, circumferential sharp dissection within the tumor-arachnoid plane rather than traction or blunt pulling, which could lead to avulsion injuries or postoperative radiculopathy [19]. In cases where the tumor directly originates from or invades the filum terminale, a safe gross total resection relies on identifying a clear boundary, sequentially coagulating, and then dividing the filum immediately superior and inferior to the mass [26]. Adhering to these micro-neurosurgical principles allowed us to achieve a 100% gross total resection rate in our cohort while ensuring complete anatomical and functional preservation of all adjacent neural structures.

Despite these challenges, consistent evidence in the literature indicates that complete resection offers durable disease control with minimal morbidity. CENETs are generally associated with an excellent prognosis, particularly following gross total resection. In the present series, no cases of tumor recurrence were observed during a mean follow-up period of approximately 60 months, supporting the favorable long-term outcomes of these lesions. Consistent with our findings, Gelabert-González et al. reported very low recurrence rates, typically ranging between 1% and 2% after complete resection [9]. Similarly, Yin et al. and Yang et al. emphasized that recurrence is uncommon and is primarily associated with subtotal resection or residual tumor [7,21]. These observations reinforce the importance of achieving complete tumor removal to minimize recurrence risk and ensure durable disease control. Although CENETs are generally regarded as nonfunctional tumors, rare cases with secretory activity have been reported. Despite the presence of neurosecretory granules, most lesions do not exhibit clinically evident catecholamine-related symptoms [8,9,27]. However, occasional cases of intraoperative hypertension or hemodynamic instability have been described, likely due to transient catecholamine release [8]. In our series, one patient developed perioperative hypertension that was successfully managed with medical therapy, suggesting possible subclinical secretory behavior. Consistent with previous reports, unrecognized catecholamine excess may be overlooked preoperatively, highlighting the importance of considering biochemical evaluation in selected patients with unexplained hypertension [27]. Histopathological examination remains the gold standard for the diagnosis of cauda equina CENETs. According to the 2022 WHO Classification of Endocrine and Neuroendocrine Tumors, these lesions are recognized within the spectrum of cauda equina neuroendocrine tumors, reflecting their distinct biological origin [3]. Previous studies have demonstrated that, unlike CENETs in other anatomical regions, those arising in the cauda equina may exhibit pan-cytokeratin (AE1/AE3) expression, supporting their unique differentiation profile [28,29]. Microscopically, these tumors characteristically display a “zellballen” architecture, composed of nests of chief cells surrounded by sustentacular cells within a rich capillary network [6,30,31]. Immunohistochemically, they are typically positive for neuroendocrine markers such as chromogranin, synaptophysin, NSE, CD56, and S-100, while lacking GFAP and EMA expression, which aids in distinguishing them from ependymomas [29]. These features collectively provide a reliable framework for differential diagnosis from other intradural tumors. Recent advances in sequencing technologies have improved the understanding of the molecular and epigenetic landscape of CENETs [3,28,32,33]. In contrast to CENETs arising in other anatomical regions, several studies have reported an absence of SDHx mutations in CENETs, suggesting a distinct genetic profile [3,28,33,34,35]. Transcription factors such as GATA3, CDX2, and TTF-1 are typically not expressed in these tumors, further supporting their unique neuroendocrine differentiation pattern. Although additional alterations, including telomerase activation, ATRX mutations, and MAML3 gene fusions, have been described, their clinical significance remains unclear and requires further validation [36,37]. Moreover, emerging epigenetic analyses indicate that CENETs may represent a distinct molecular subgroup, differing from both systemic CENETs and other neuroendocrine neoplasms [33].

CENETs are overwhelmingly non-functional; direct mechanical manipulation during surgical dissection can trigger a sudden release of stored intracellular catecholamines. This phenomenon was observed in Case 4, where the patient experienced a transient intraoperative hypertensive crisis strictly during tumor mobilization, which resolved rapidly following total resection. Because this was an unexpected intraoperative surge rather than a pre-planned medical optimization, immediate rescue therapy with a short-acting intravenous beta-blocker was titrated by the neuroanesthesia team. However, we must emphasize that for any patient with suspected or biochemically proven catecholamine excess preoperatively, beta-blocker monotherapy is strictly contraindicated due to the risk of unopposed $\alpha_1$-mediated vasoconstriction [38]. This experience highlights that while acute surges during tumor handling can be safely managed with rapid rescue anesthetics, preoperative biochemical screening should be considered if atypical cardiovascular fluctuations are present before surgery.

This study has several limitations. First, its retrospective design and relatively small sample size limit the generalizability of the findings. Second, the single-center nature of the study may introduce selection bias. In addition, the preoperative diagnostic workup did not include biochemical screenings (such as plasma or urinary fractionated metanephrines) or functional neuroendocrine imaging (such as DOTATATE or MIBG scintigraphy). Because CENETs present with highly nonspecific radiological features on routine MRI, they are rarely included in the initial pre-surgical differential diagnosis, which is typically dominated by ependymomas and nerve sheath tumors; consequently, these specialized evaluations were not clinically prompted. Postoperatively, advanced metabolic workups and germline genetic testing could not be systematically performed due to socioeconomic factors, as these high-cost tests were not covered by public health insurance for completely resected, histologically low-grade sporadic lesions, and patients declined out-of-pocket payment. Finally, although the follow-up period was adequate, longer-term data may be necessary to fully assess recurrence patterns and long-term outcomes.

## 6. Conclusions

CENETs are rare intradural extramedullary tumors with a generally favorable prognosis. Although preoperative diagnosis remains challenging due to nonspecific clinical and radiological findings, characteristic vascular imaging features may provide useful diagnostic clues. In our series, gross total resection was achieved in all patients, resulting in excellent neurological outcomes and no recurrence during follow-up. Complete surgical resection remains the cornerstone of management, offering durable disease control with low morbidity.

## Figures and Tables

**Table 1:** Clinical and Radiological Characteristics of 9 Patients with Intradural Extramedullary Tumors.

No	Age	Sex	Comorbidities	PAIN	RADICULOPATY	HİPOESTESİA	INCONTINANS	Symptom Duration (months)	Preop (McCormick Grade)	MRI Level (L/S)	Craniocaudal Size (mm)	Anteroposterior Size (mm)	Transverse Size (mm)	T1	T2	Post-Contrast T1-Weighted Imaging (T1WI)	Flow Voids (Present/Absent)	Cystic Element	Hemorrhagic Features	Hemorrhagic Cap Sign (Present/Absent)	Eccentric Vessel Sign (Present/Absent)	Dumbbell Sign (Present/Absent)	Tadpole Sign (Present/Absent)
1	47	F	-	No	Yes	Yes	No	4	2	L4	9	10	11	ISOINTENSE	ISOINTENSE	homojen	-	-	-	-	present	-	present
2	52	M	DM	Yes	No	No	No	5	1	L3	19	10	14	ISOINTENSE	ISOINTENSE	homojen	-	-	-	-	-	-	present
3	38	M		Yes	Yes	Yes	No	24	2	L2	38	12	18	ISOINTENSE	HYPERINTENSE	homojen	present	-	-	-	present	-	-
4	57	M	-	Yes	No	No	No	6	1	S1	26	15	18	ISOINTENSE	ISOINTENSE	heterojen	present	-	present	present	-	-	present
5	44	F	HBV	Yes	No	No	No	4	1	L3	18	11	13	ISOINTENSE	HYPERINTENSE	heterojen	present	present	-	-	present	-	-
6	67	F	-	Yes	Yes	Yes	Yes	3	2	L1-2	46	9	11	ISOINTENSE	ISOINTENSE	homojen	present	-	present	-	present	-	present
7	54	M	-	Yes	No	No	No	6	1	L2	17	12	12	ISOINTENSE	HYPERINTENSE	homojen	-	-	present	present	present	-	-
8	57	F	-	Yes	No	No	No	12	1	L4-5	14	12	15	ISOINTENSE	ISOINTENSE	homojen	present	-	present	present	present	-	-
9	45	M	-	No	Yes	Yes	No	3	2	L3	20	12	13	ISOINTENSE	HYPERINTENSE	heterojen	-	present	present	present	-	-	-

**Table 2:** Summary of Clinical, Radiological, Surgical, and Outcome Data (*n* = 9).

Category	Variable	Result
**Demographics**	Age (years)	Mean 51.2 (Range: 38–67)
	Sex	5 Male/4 Female (1.25:1)
	Comorbidities	22.2% (2/9): DM (*n* = 1), HBV (*n* = 1)
**Clinical Presentation**	Pain	77.8% (7/9)
	Radiculopathy	44.4% (4/9)
	Hypoesthesia	44.4% (4/9)
	Paresthesia	0% (0/9)
	Incontinence	11.1% (1/9)
	Symptom Duration	Mean 7.4 months (Range: 3–24)
**Preoperative Status**	McCormick Grade I	55.6% (5/9)
	McCormick Grade II	44.4% (4/9)
	McCormick Grade ≥ III	0%
**Tumor Characteristics**	Location	100% Intradural-Extramedullary
	Vertebral Levels	L3 (33.3%), L2 (22.2%), L4/L4-5 (22.2%), S1 (11.1%)
	Craniocaudal Size	Mean 23.3 mm (9–46)
	Anteroposterior Size	Mean 11.4 mm (9–15)
	Transverse Size	Mean 13.9 mm (11–18)
**MRI Findings**	T1 Signal	100% Isointense
	T2 Signal	Isointense 55.6%, Hyperintense 44.4%
	Contrast Enhancement	Homogeneous 66.7%, Heterogeneous 33.3%
**Imaging Features**	Flow Voids	55.6% (5/9)
	Hemorrhagic Features	55.6% (5/9)
	Hemorrhagic Cap Sign	44.4% (4/9)
	Eccentric Vessel Sign	66.7% (6/9)
	Tadpole Sign	44.4% (4/9)
	Cystic Elements	22.2% (2/9)
	Dumbbell Sign	0%
	Osseous Remodeling	0%
**Surgical Data**	Time to Surgery	Median 27 days (3-913)
	Approach	Laminectomy 44.4%, Hemilaminectomy 44.4%, Laminoplasty 11.1%
	Neural Preservation	100%
	Gross Total Resection	100% (9/9)
**Early Outcomes**	Improved	44.4% (4/9)
	Stable	55.6% (5/9)
	Worsened	0%
	Early Complications	0%
**Pathology (IHC)**	Chromogranin	100% Positive
	Synaptophysin	100% Positive
	S100	88.9% Positive
	Ki-67 Index	Mean 7.0% (Range: 2–15%)
**Postoperative Course**	Hospital Stay	Mean 4.3 days (Range: 3–7)
	Adjuvant Therapy	0%
**Long-term Outcomes**	Follow-up Duration	Mean 59.6 months (12–134)
	Recurrence	0%
	Late Complications	11.1% (1/9: cephalalgia/hypertension)

**Table 3:** Surgical, and Histopathological Characteristics of 9 Patients with Intradural Extramedullary Tumors.

No	Time from Diagnosis to Surgery (days)	Surgical Approach (Total Laminectomy-1/Hemilaminectomy-2/Laminoplast-3)	Filum/Root Status (Sacrificed/Preserved)	Resection (GTR/STR/Bx)	Early Neurological Outcome (Improved/Stable/Worsened)	Chromogranin	Synaptophysin	S100	Ki-67%	Post-Surgery Complications	Length of Hospital Stay (days)	Adjuvant Therapy	Follow-Up (months)	Complications (Hypertension, Cephalalgia, etc.)	Recurrence
1	3	2	PRESERVED	GTR	Improved	POSITIVE	POSITIVE	POSITIVE	6%	-	3 DAYS	No	24	No	No
2	21	2	PRESERVED	GTR	Stable	POSITIVE	POSITIVE	POSITIVE	15%	-	4 DAYS	No	46	No	No
3	120	3	PRESERVED	GTR	Improved	POSITIVE	POSITIVE	POSITIVE	8%	-	4 DAYS	No	30	No	No
4	27	1	PRESERVED	GTR	Stable	POSITIVE	POSITIVE	POSITIVE	4%	-	3 DAYS	No	35	Yes	No
5	80	1	PRESERVED	GTR	Stable	POSITIVE	POSITIVE	NR	8%	-	4 DAYS	No	106	No	No
6	913	1	PRESERVED	GTR	Improved	POSITIVE	POSITIVE	POSITIVE	6%	-	7 DAYS	No	131	No	No
7	185	1	PRESERVED	GTR	Stable	POSITIVE	POSITIVE	POSITIVE	7%	-	3 DAYS	No	134	No	No
8	6	2	PRESERVED	GTR	Stable	POSITIVE	POSITIVE	POSITIVE	7%	-	4 DAYS	No	18	No	No
9	5	2	PRESERVED	GTR	Improved	POSITIVE	POSITIVE	POSITIVE	2%	-	7 DAYS	No	12	No	No

## Data Availability

The datasets generated during and/or analyzed during the current study are available from the corresponding author on reasonable request.
